# Gender Identity in Autism: Sex Differences in Social Affiliation with Gender Groups

**DOI:** 10.1007/s10803-018-3590-1

**Published:** 2018-04-28

**Authors:** Kate Cooper, Laura G. E. Smith, Ailsa J. Russell

**Affiliations:** Centre for Applied Autism Research, Department of Psychology, University of Bath, Claverton Down, Bath, BA2 7AY UK

**Keywords:** Gender, Social identity, Self-esteem, Sex differences, Autism, Adults

## Abstract

High rates of gender variance have been reported in autistic people, with higher variance in autistic females than males. The social component of gender identity may be affected, with autistic females experiencing lower identification with and feeling less positively about their gender groups than controls. We measured gender identification, gender self-esteem, and aspects of gender expression (masculinity and femininity) in autistic natal males and females, and controls (*N* = 486). We found that autistic people had lower gender identification and gender self-esteem than controls, and autistic natal females had lower gender identification than autistic natal males and natal female controls. In conclusion, autistic people, particularly natal females, had lower social identification with and more negative feelings about a gender group.

## Introduction

Autism spectrum disorder (ASD, referred to as “autism” in this paper) is characterised by difficulties with social communication, alongside repetitive, restricted behaviours, interests or activities, as outlined in The Diagnostic and Statistical Manual of Mental Disorders (5th ed.; DSM–5; American Psychiatric Association 2013). Autism is reported to affect around 1% of the population, with a disproportionately higher number of natal males diagnosed with the condition compared to natal females (Baird et al. 2006). Some recent research has found sex differences in the autism phenotype (e.g. Mandy et al. 2012; Lai et al. 2011; Hiller et al. 2014) but results have been mixed regarding the nature of such sex differences. For example, a systematic review and meta-analysis found boys had higher levels of repetitive behaviours after the age of 6 years but that there were no sex differences in social communication across the lifespan in autism (Van Wijngaarden-Cremers et al. 2014).

The extreme male brain theory of autism (Baron-Cohen 2002) proposes that the cognitive profile of autistic people is characteristically ‘male’ which can be “defined psychometrically as those individuals in whom systemising is significantly better than empathising” (Baron-Cohen 2002, p. 248). This profile has been labelled as male due to evidence of sex differences in these cognitive abilities in the general population, with women outperforming men in empathising and men outperforming women in systemising skills (although these sex differences are modulated by cultural norms and individual motivation^1^). Physiological sex differences also support this theory; Ecker et al. (2017) found that natal females whose brain anatomy was more similar to typical natal males, based on measures of cortical thickness, were three times more likely to have autism than natal females with more typically female brain anatomy. Therefore autism has been described as a case of the extreme male brain. It is possible that receiving a diagnosis of autism, with traits perceived to be male, has implications for how autistic natal females feel about their biological sex and gender. There is some research evidence to suggest that autistic natal females may not identify as readily with conventional female gender norms. Qualitative research findings suggest that autistic girls and women prefer socialising with boys and men rather than women (Bargiela et al. 2016; Cridland et al. 2014), and do not readily identify with the construct of femininity (Kanfiszer et al. 2017). Gender variance is defined as gender identity or gender expression which does not conform to traditional masculine or feminine gender norms (see Table 1 for definitions relating to gender in this paper), and sex differences in the prevalence of gender variance in autism have been reported. Studies have reported 22% (Dewinter et al. 2017) and 33% (Bejerot and Erikson 2014; George and Stokes 2017) of autistic natal females are gender variant compared to 8% (Dewinter et al. 2017) and 22% (George and Stokes 2017) of natal males. Another study found no sex differences in gender variance in autistic children (Strang et al. 2014), although this result could be confounded by unequal group sizes, as this study compared 24 natal females to 123 natal males.

**Table 1:** Definitions of key terms

Term	Definition
Sex	The biological characteristics (including chromosomes, genitals and hormones) that categorise humans as male or female
Gender	The socially constructed behavioural norms for each sex
Gender identity	The self-defined label an individual gives their gender based on their own experience and felt-sense of identity e.g., male, female, non-binary, gender neutral, gender fluid, genderqueer
Gender congruence	When gender identity is congruent with biological sex
Gender variance	Gender identity or gender expression which does not conform to masculine or feminine gender norms, for example having a gender identity different to one’s biological sex, or not defining oneself within the gender binary of ‘male/female’
Gender group	A social group of individuals who consider themselves to have the same or similar gender identities
Gender expression	The way in which an individual expresses their gender identity, the most common gender expressions often being classified as either “masculine” or “feminine”
Masculinity	The gender norms, roles and traits traditionally associated with the male sex
Femininity	The gender norms, roles and traits traditionally associated with the female sex
Gender identification	Psychological attachment to a gender group, typically ‘men’ or ‘women’
Gender self-esteem	How positively (or negatively) an individual views their gender group

When an individual is distressed by the experience of their gender identity opposing their biological sex, this is termed gender dysphoria. Gender dysphoria is characterised in the *Diagnostic and Statistical Manual of Mental Disorders* (5th ed.; *DSM–5;* American Psychiatric Association 2013) by the presence of two of the following criteria; incongruence between one’s sex and expressed gender; a strong desire to transition away from one’s current sex; a strong desire to transition to the other sex; a desire to belong to and/or be treated as belonging to a different gender group; a conviction that one has the typical feelings and reactions of the other gender. Studies identifying autism diagnoses in those attending gender clinics have found rates of autism diagnoses between 5.5–26% (Pasterski et al. 2014; Kaltiala-Heino et al. 2015), notably higher than the 1% prevalence of autism more generally (e.g. Baird et al. 2006).

High rates of gender variance have been reported in both autistic natal males and females, suggesting that while natal females are more affected, autistic people as a group are more likely to express gender variance than typically developing controls. Studies investigating gender variance have found autistic people display less stereotypically male role behaviour compared to sex-matched typically developing controls (Stauder et al. 2011; Bejerot and Eriksson 2014). Studies using a range of methods have provided evidence for gender variance and gender dysphoria in autism across the sexes including case studies (Williams et al. 1996; Landen and Ramussen 1997; Mukaddes 2002; Perera et al. 2003; Kraemer et al. 2005; Tateno et al. 2008; Jacobs et al. 2014; Lemaire et al. 2014); studies identifying high rates of gender variance in participants with an autism diagnosis (Stauder et al. 2011; Bejerot and Eriksson 2014; Strang et al. 2014; Janssen et al. 2016; May et al. 2017; Dewinter et al. 2017; Kanfiszer et al. 2017); and studies of elevated levels of autistic traits or diagnoses in those attending gender clinics (de Vries et al. 2010; Jones et al. 2012; Pasterski et al. 2014; Skagerberg et al. 2015; Kaltiala-Heino et al. 2015; VanderLaan et al. 2015; Shumer et al. 2016); and narrative and systematic reviews of the literature (van Schalkwyk et al. 2015; Glidden et al. 2016; Van Der Miesen et al. 2016).

Gender dysphoria and gender variance can be conceptualised as being parts of the spectrum of gender identity, with all individual gender identities belonging somewhere on this spectrum. *Gender identification* is a distinct construct from gender identity, and relates to one’s social identity as a member of a gender group. We define gender identification (in the terms of social identity theory; Tajfel and Turner 1979) as psychological affiliation to a gender group (cf. Tajfel 1981; Table 1), for example, someone with the gender identity ‘woman’ strongly identifying with other women due to their shared gender group. The gender groups which are most dominant are those of ‘men’ and ‘women’, however gender is increasingly being conceptualised as a spectrum, rather than a binary construct. Therefore there are numerous gender groups an individual could belong to, and some individuals may consider that they do not belong to a gender group at all, but rather are “gender free”. It may well be that autistic people, who a more likely to be gender diverse, are less likely to feel a sense of affiliation to any gender group, be that “men”, “women”, “transgender people”, “nonbinary people” etc. This is because autistic people are more likely to belong to minority gender groups, with fewer members and therefore less access to other in-group members, which may reduce a sense of affiliation within groups. However it is also possible that individuals belonging to minority gender groups will affiliate more strongly and positively with in-group members in order to mitigate the effect of belonging to a stigmatised group, and so more research is needed to understand the relationship between autism, gender variance and social affiliation with a gender group. Research recruiting autistic participants, who are more likely to be gender diverse, should therefore measure social affiliation with *any* gender group, without constraining responses to gender normative groups, in order to accurately reflect social affiliations in a gender diverse community.

Positive feelings about a gender group, or *gender self-esteem*, can be measured through self-report scales of group self-esteem (e.g. Luhtanen and Crocker 1992). For example, the gender self-esteem of an individual who identifies as non-binary would relate to how positively they view the group ‘non-binary people’. No quantitative research has investigated how strongly autistic people identify with a gender group, and how positively they feel about a gender group.

A sense of social affiliation with a gender group has been shown to be positively associated with psychological well-being in typically developing people (Good and Sanchez 2010). This finding extends to those with varied gender identities; a study with typically developing transgender women showed that positive feelings about gender identity were correlated with improved psychological well-being (Sanchez and Vilain 2009). In autistic people a sense of social affiliation with other autistic people has been found to be related to improved psychological well-being (Cooper et al. 2017). Furthermore, George and Stokes (2018) found that autistic individuals with gender dysphoric traits had poorer psychological well-being (higher stress, depression, and anxiety and lower well-being) than autistic people without these traits. Therefore social affiliation with a gender group presents as an important construct to measure in autistic people, a group high in gender diversity, who are known to be vulnerable to mental health problems (Hofvander et al. 2009).

In terms of sex differences in gender identification in the general population, natal females and males tend to have equivalent scores in terms of gender identification, i.e. strength of affiliation with gender identity (e.g. Schmader 2002). Furthermore, natal females tend to feel more positively about their gender group (have higher gender self-esteem) than natal males (Barker 2009), or have equivalent gender self-esteem to natal males (Foels and Tomcho 2005). However, no studies to date have investigated how gender and related constructs are conceptualised by autistic people, and because of this knowledge gap, it is not known if there are sex differences in gender identification and gender self-esteem in autistic people.

### Aims and Hypotheses

Given the evidence suggesting high rates of gender variance and dysphoria in autism, the purpose of this research was to investigate whether having autism affects the extent to which natal males and females identify with a gender group, and how positively they feel about these gender groups. In this article, we test the proposition that the autistic natal females will have higher self-perceived gender variance than autistic natal males (gender identity different to biological sex, and masculinity and femininity ratings incongruent with biological sex), which will affect how attached they feel to a gender group (gender identification), and the extent to which they feel positively about their gender group (gender self-esteem).

We hypothesised that autistic participants would have lower gender identification and gender self-esteem than typically developing controls. We also hypothesised that participants who are not gender congruent would have lower gender identification and gender self-esteem than gender congruent participants. Furthermore, autistic natal females would consider themselves to be significantly more masculine and less feminine than typically developing natal females. Finally, we predicted sex differences within the autism group; autistic natal females would have lower gender identification and gender self-esteem than autistic natal males.

## Method

### Participants and Design

This study had a 2 (sex: male versus female) × 2 (autism: autism vs. typically developing; TD controls) × 2 (gender congruence: congruent vs. incongruent) factorial design. We recruited four groups of participants online (*N* = 486): autistic females (*n* = 101), autistic males (*n* = 118), TD females (*n* = 153) and TD males (*n* = 114). For brevity, the names of these groups refer to participants’ assigned biological sex at birth, not their self-defined gender group. We recruited participants from a variety of sources such as online forums for the autism community, autism organisation websites and group networks. Controls were recruited through social media, university networks and personal contacts. Participants were aged between 16 and 80 years and without known or suspected intellectual disability. Participants were included in the autism group if they reported that they had received a diagnosis of any Autism Spectrum Disorder (i.e., Asperger’s syndrome, high functioning autism, autism, atypical autism, pervasive developmental disorder (PDD) and PDD-not otherwise specified), referred to in this article using the term autism or ASD. The source of this diagnosis was not recorded or validated, but only participants who reported that they had received a formal diagnosis of ASD were included in the analyses. While an independent assessment of autism diagnosis is important in research to confirm diagnostic group membership, this online sampling method allowed us to reach a wider cross-section of autistic people. Similar methods have been successfully been utilised for other large online studies (e.g., Kenny et al. 2015).

Demographics information regarding; mental health diagnosis, educational achievement, age, age of autism diagnosis (for autistic participants), and sexual orientation identity was collected (Table 2). Participants also completed questionnaires regarding autism social identity and collective self-esteem, depression, anxiety and self-esteem used in another study (Cooper et al. 2017), although they completed separate measures for each paper, with the exception of the demographic measures.

**Table 2:** Demographic information for autistic female (n = 101), autistic male (n = 118), TD female (n = 153), and TD male (n = 114) participants

	Autistic female	Autistic male	TD female	TD male
Mean age (SD)	30.38 (12.4)	33.2 (12.53)	35.88 (11.5)	32.02 (13.0)
Mean age at diagnosis (SD)	24.82 (14.05)	27.88 (14.76)	–	–
University graduate (%)	56 (55)	52 (44)	118 (78)	91 (80)
Mental health diagnosis *n* (%)	72 (71)	69 (59)	45 (30)	24 (21)
Heterosexual identity *n* (%)	32 (31)	74 (63)	127 (84)	90 (79)
Gender transition *n* (%)	19 (19)	5 (4)	5 (3)	3 (3)
*Gender identity*				
Male *n* (%)	7 (7)	**105 (89)**	2 (1)	**108 (95)**
Female *n* (%)	**67 (66)**	4 (3)	**149 (97)**	2 (2)
Other *n* (%)	27 (27)	9 (8)	2 (1)	4 (4)

Bold values indicate the gender identity aligned with participants’ birth sex

Test materials and information sheets were adapted to ensure accessibility to autistic people, by ensuring that the wording of questionnaires was clear and no metaphors were used. Ethical approval was obtained from the University of Bath ethics committee (approval number: 14–023) and fully informed consent was obtained from all participants.

### Measures

#### Measures of Gender Identity and Transition

Participants provided the following information: biological sex assigned at birth; gender identity (male, female, or ‘other’), gender transition (defined as a positive response to the question “are you considering, or taking action, to change from your physical sex at birth to your gender identity?”). The gender identity item was used to generate the ‘gender congruence’ variable. Participants who reported a gender identity which was the same as their birth sex were labelled as ‘gender congruent’, and those with a gender identity different to their birth sex were labelled ‘gender incongruent’.

#### Masculinity and Femininity

Self-reported masculinity and femininity was measured to investigate an individual’s gender identity in relation to traditional gender norms, which may not have been evident from their response to the gender identity or gender dysphoria items. Two single items were used in order to reduce the burden on participants resulting from the addition of another questionnaire. Participants responded to the two items “how masculine would you rate yourself?” and “how feminine would you rate yourself?” on a 7-point Likert-type scale, with 1 representing “not at all” and 7 “very much”.

#### Gender Identification

The measure of social identification with a gender group has three items. It is based on a validated and reliable social identification scale by Doosje et al. (1995). The items were “I identify with other people of the same gender as myself”, “I am glad to be the gender I am” and “I feel strong ties with members of the same gender as myself”. One item was removed from the original scale; “I see myself as [gender identity]” so that the measure exclusively investigated the affiliative and affective components of gender identification, rather than focusing on clarity of gender identity. The scale used the term “gender” to allow participants from a wide range of gender groups to participate, rather than forcing participants to respond based on their sex assigned at birth. This was thought to be important for this group of participants given the wide range of gender identities reported by autistic people. Thus this measure of gender identification captured the extent to which participants’ felt affiliated with and derived positive affect from a gender group. Participants responded to these items on a 7-point Likert-type scale where 1 = strongly disagree and 7 = strongly agree. The scale had good internal consistency (α = .81) in the current study.

#### Gender Self-Esteem

A reliable and valid measure of gender self-esteem (how positively an individual sees their gender group) was given to participants. For this purpose, we used the collective self-esteem scale (Luhtanen and Crocker 1992). Two of the four subscales which measure private collective self-esteem (how positively the individual thinks of their gender group) and public collective self-esteem (how positively the individual thinks others consider their gender group) were used, resulting in an 8-item scale with good internal consistency in this study (α = .89). Items included “overall, my gender group is considered good by others”, and “I feel good about the gender group I belong to.” Participants responded to these items on a 7-point Likert-type scale where 1 = strongly disagree and 7 = strongly agree.

### Analysis Plan

A series of Chi square analyses were used. The first tested for associations between the two categorical gender-related measures gender identity and gender transition. The next Chi square analyses tested for associations between the gender related measures and (a) autistic and typically developing groups and (b) males and females within the autism group.

We used hierarchical multiple regression using categorical predictor ‘dummy’ variables for the categorical groups: sex (male = 0 and female = 1), autism (TD = 0 and autism = 1) and gender congruence (gender congruent = 0 and gender incongruent = 1) at Step 1, using the interaction terms sex*autism, autism*gendercongruence and sex*gendercongruence at Step 2, and the three way interaction term at Step 3 to investigate group differences on the main experimental variables. We used multiple regression in place of factorial MANOVA due to unequal cell sizes (Tabachnik and Fidell 2007).

## Results

### Gender Congruence and Autism

Autistic participants were significantly more likely to be gender incongruent (defined as selecting an ‘other’ gender label or a gender label different to biological sex) than TD controls, χ^2^(1, *N* = 485) = 45.98, *p* < .001. Further, autistic females were significantly more likely to be gender incongruent than autistic males χ^2^(1, *N* = 219) = 150.24, *p* < .001. See Table 2. Examples of ‘other’ gender labels that participants chose included ‘androgynous’, ‘alien’, ‘gender neutral’, ‘gender fluid’ and ‘not sure’.

### Gender Transition and Autism

Autistic participants were significantly more likely to have or be planning a gender transition than TD participants, χ^2^(1, *N* = 485) = 12.40, *p* < .001, and autistic females were significantly more likely to have or be planning gender transition than autistic males, χ^2^(1, *N* = 219) = 11.85, *p* = .001.

### Gender Transition and Gender Congruence

Gender incongruent participants were significantly more likely to have or be planning a gender transition than gender congruent participants, χ^2^(1, *N* = 486) = 189.97, *p* < .001.

### Gender Identification and Gender Self-Esteem

We conducted two hierarchical multiple regressions, entering the factors sex (male or female), autism (Autism or TD), and gender congruence (gender congruent or gender incongruent) at Step 1, and the sex*autism, sex*gendercongruence and autism*gendercongruence interaction terms at Step 2, and the three way interaction term at Step 3, predicting gender identification and gender esteem, respectively. See Tables 3 and 4 for means, SDs and correlations for these variables. In the first model, predicting gender identification, we found that autism was significantly negatively associated with gender identification, β = − .399, *t*(482) = − 10.07, *p* < .001, with autistic participants (*M* = 4.18, *SD* = 1.32) scoring significantly lower than TD controls (M = 5.48, SD = 1.03). Gender congruence was negatively associated with gender identification, β = − .298, *t*(482) = − 7.54, *p* < .001, with gender congruent participants (*M* = 5.09, *SD* = 1.12) having significantly higher gender identification than gender incongruent participants (*M* = 3.42, *SD* = 1.34). There was no overall difference between males (*M* = 4.81, *SD* = 1.27) and females (*M* = 4.97, *SD* = 1.39) on gender identification, β = − .046, *t*(482) = − 1.20, *p* = .229.

**Table 3:** Means and SDs for gender congruent (n = 429) and gender incongruent (n = 57) participants

Group	*n*	1. Gender identification		2. Gender self-esteem		3. Masculinity		4. Femininity	
		*M*	SD	*M*	SD	*M*	SD	*M*	SD
*Gender congruent*									
Autistic natal female	67	4.30	1.20	17.69	3.53	3.54	1.42	4.27	1.38
Autistic natal male	105	4.50	1.20	19.44	4.18	4.68	1.28	2.63	1.16
TD natal female	149	5.63	0.99	20.83	3.60	2.58	1.33	5.19	1.29
TD natal male	108	5.41	0.97	21.51	3.69	4.98	1.18	2.88	1.47
*Gender incongruent*									
Autistic natal female	34	3.43	1.42	15.62	4.50	4.56	1.35	2.65	1.18
Autistic natal male	13	2.92	1.25	13.81	4.91	3.08	1.26	4.15	1.28
TD natal female	4	4.59	0.92	15.13	2.53	4.50	0.58	3.50	1.00
TD natal male	6	3.67	0.92	16.58	3.47	2.83	1.94	4.17	2.32

**Table 4:** Correlations for gender congruent (n = 429) and gender incongruent (n = 57) participants

Group	Measure	1.	2.	3.	4.
		*r*	*r*	*r*	*r*
*Gender congruent*	1. Gender identification	–	.568**	− .114*	.238**
	2. Gender self-esteem	–	–	− .028	.074
	3. Masculinity	–	–	–	− .684**
	4. Femininity	–	–	–	–
*Gender incongruent*	1. Gender identification	–	.701**	.260	− .242
	2. Gender self-esteem	–	–	.128	− .254
	3. Masculinity	–	–	–	− .453**
	4. Femininity	–	–	–	–

*Correlation is significant at the 0.05 level

**Correlation is significant at the 0.01 level

The variables at Step 1 explained a significant amount of variance in gender identification, *R*^2^ = .316, *F*(3, 482) = 74.38, *p* < .001.

At Step 2, there was a significant 2-way interaction between sex and autism, β = − .080, *t*(479) = − 2.01, *p* < .05, and between sex and gender congruence, β = .132, *t*(479) = 2.02, *p* < .05, but there was no 2-way interaction between autism and gender congruence, β = .031, *t*(479) = .408, *p* = .68. The second step did not significantly increase the variance explained at Step 1, *R*^2^ch. = .010, *F*ch. (3, 479) = 2.38, *p* = .069. At Step 3, the three way interaction term was not a significant predictor of gender identification, β = .002, *t*(478) = .03, *p* = .98, and did not significantly contribute to the variance explained, *R*^2^ch. = .000, *F*ch. (1, 478) = .001, *p* = .98.

**Fig. 1:**
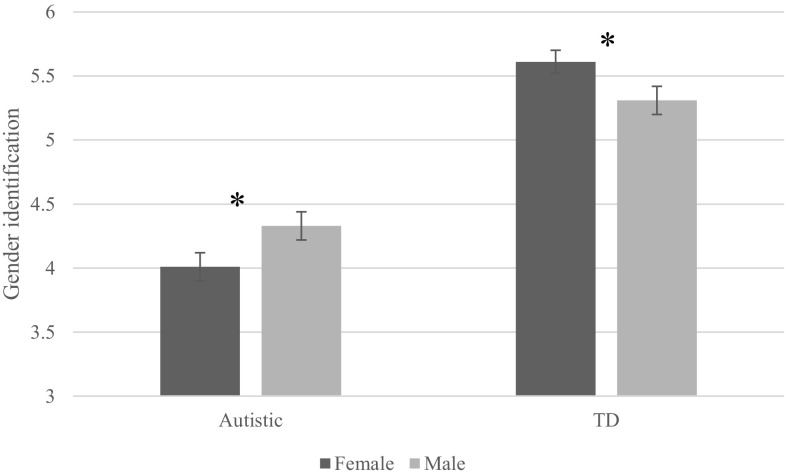
Differential effects of sex on gender identification between autistic and typically developing participants

Simple slopes analysis for the sex*autism interaction revealed that TD females (*M* = 5.61, *SD* = 1.00) had significantly higher gender identification than TD males (*M* = 5.31, *SD* = 1.04), β = − .368, *t*(482) = -6.47, *p* < .001 (see Fig. 1). However, the inverse was found for autistic participants; autistic females (*M* = 4.01, *SD* = 1.33) had significantly lower gender identification than autistic males (*M* = 4.33, *SD* = 1.30), β = − .597, *t*(482) = -10.75, *p* < .001.

Simple slopes analysis for the sex*gendercongruence interaction revealed that there was no significant effect of sex on gender identification between natal males (*M* = 3.16, *SD* = 1.18) and natal females (*M* = 3.55, *SD* = 1.41) in gender incongruent participants, β = .148, *t*(482) = 1.15, *p* = .25. However there was a significant effect of sex on gender identification in gender congruent participants, with gender congruent females having significantly higher gender identification (*M* = 5.22, *SD* = 1.22) than gender congruent males (*M* = 4.96, *SD* = 1.18), β = .097, *t*(482) = 2.21, *p* < .05.

In the second model, predicting gender self-esteem, we found that autism was a significant predictor, β = − .274, *t*(482) = − 6.43, *p* < .001, with autistic people (*M* = 17.98, SD = 4.41) scoring significantly lower than TD controls (*M* = 20.93, *SD* = 3.74). Sex was also a significant predictor, β = − .094, *t*(482) = 2.27, *p* < .05, with males (*M* = 20.02, *SD* = 4.40) scoring higher than females (*M* = 19.21, *SD* = 4.21). Incongruent gender was a predictor, β = − .281, *t*(482) = − 6.58, *p* < .001, with participants with a congruent gender (*M* = 20.17, *SD* = 3.97) scoring higher than those with an incongruent gender (*M* = 15.27, *SD* = 4.39). The variables at Step 1 explained a significant amount of variance in gender self-esteem, *R*^2^ = .205, *F*(3, 482) = 41.44, *p* < .001. At Step 2, neither the autism*sex nor the autism*gendercongruence interaction terms significantly predicted gender self-esteem, however the sex*gendercongruence interaction term was a predictor β = − .281, *t*(479) = 2.10, *p* < .05. The model at Step 2 did not explain a significantly higher proportion of the variance than the model at Step 1, *R*^2^ch. = .011, *F*ch. (3, 479) = 2.19, *p* = .089. At Step 3, the three-way interaction term was not a significant predictor, β = .123, *t*(478) = 1.51, *p* = .13, *R*^2^ch. = .004, *F*ch. (1, 478) = .133, *p* = .13.

Simple slopes analysis for the sex*gendercongruence interaction revealed that there was no significant effect of sex on gender self-esteem in gender incongruent participants, β = .102, *t*(482) = .782, *p* = .44, although natal males (*M* = 14.68, *SD* = .92) scored lower than natal females (*M* = 15.60, *SD* = .65). Nor was there a significant effect of sex on gender self-esteem in the gender congruent participants, β = − .074, *t*(482) = -1.64, *p* = .102, although the inverse pattern to gender incongruent participants was apparent from the means, with males (*M* = 20.49, *SD* = .28) scoring higher than females (*M* = 19.86, *SD* = .27).

### Masculinity and Femininity

We conducted two hierarchical multiple regressions, predicting self-reported masculinity and femininity respectively. We entered the factors sex (male or female), autism (Autism or TD), and gender congruence (gender congruent or gender incongruent) at Step 1, the sex*autism, sex*gendercongruence and autism*gendercongruence interaction terms at Step 2, and the three way interaction term at Step 3. See Tables 3 and 4 for means, SDs and correlations. In the model predicting masculinity, at Step 1, autism was a significant predictor β = .125, *t*(482) = 3.01, *p* < .01, with autistic participants having significantly higher masculinity (*M* = 4.22, *SD* = 1.45) than typically developing participants (*M* = 3.58, *SD* = 1.73). Sex was also a significant predictor, with male participants (*M* = 4.68, *SD* = 1.35) reporting significantly higher masculinity than female participants (*M* = 3.13, *SD* = 1.53), β = .47, *t*(482) = 11.58, *p* < .001. Gender congruence was not a significant predictor, β = .05, *t*(482) = 1.24, *p* = .21. The model at Step 1 accounted for a significant proportion of the variance in masculinity, *R*^2^ = .246, *F*(3, 482) = 52.54, *p* < .001. At Step 2, the autism*sex interaction term was a significant predictor, β = .176, *t*(479) = 4.59, *p* < .001, as was the sex*gendercongruence interaction term, β = .451, *t*(479) = 7.14, *p* < .001. The autism*gendercongruence interaction term was not a significant predictor, β = − 0.19, *t*(479) = − 0.258, *p* = .80. The model at Step 2 explained a significantly higher proportion of the variance than the model at Step 1, *R*^2^ch.= .126, *F*ch. (3, 479) = 32.17, *p* < .001. At Step 3, the three way interaction term was not a significant predictor of masculinity, β = − .108, *t*(478) = − 1.48, *p* = .14, and the model did not account for a significantly higher proportion of the variance, *R*^2^ch. = .003, *F*ch. (1, 478) = 2.19, *p* = .14.

**Fig. 2:**
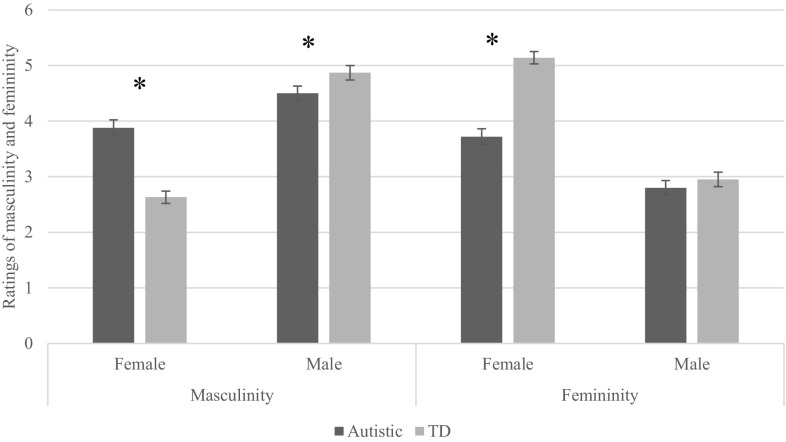
Differential effects of sex on masculinity and femininity ratings between autistic and typically developing participants

Simple slopes analysis showed that there was a significant effect of having autism on masculinity in females, with autistic females (*M* = 3.88, *SD* = .14) having significantly higher masculinity than typically developing females (*M* = 2.63, *SD* = .11), β = .381, t(482) = 7.12, p < .001. The inverse was found in males, with autistic males (*M* = 4.50, *SD* = .13) having significantly lower masculinity than typically developing males (*M* = 4.87, *SD* = .13), β = − .683, *t*(482) = − 13.19, *p* < .001 (see Fig. 2).

Simple slopes analysis revealed that there was a significant effect of sex on masculinity in gender incongruent participants, β = .473, *t*(482) = 4.13, *p* < .001, with females (*M* = 4.53, *SD* = .35) reporting higher levels of masculinity than males (*M* = 2.96, *SD* = .32). There was also a significant effect of sex on masculinity in gender congruent participants in the opposite direction, β = − .596, *t*(482) = − 15.14, *p* < .001, with males (*M* = 4.83, *SD* = .09) reporting higher levels of masculinity than females (*M* = 3.06, *SD* = .10).

In the model predicting femininity, autism was a significant predictor β = − .20, *t*(482) = − 5.04, *p* < .001, with autistic participants having significantly lower femininity than TD participants. Sex was also a significant predictor, β = − .489, *t*(482) = − 12.74, *p* < .001, with female participants (*M* = 4.58, *SD* = 1.56) having significantly higher femininity than male participants (*M* = 2.87, *SD* = 1.41). Gender congruence was a significant predictor, β = − .114, *t*(482) = − 2.89, *p* < .01, with gender congruent participants (*M* = 3.84, *SD* = 1.72) having significantly higher femininity than gender incongruent participants (*M* = 3.21, *SD* = 1.48). The variables at Step 1 accounted for a significant proportion of the variance in femininity, *R*^2^ = .314, *F*(3,482) = 73.64, *p* < .001. At Step 2, the sex*autism interaction term was a significant predictor of femininity, β = − .099, *t*(479) = − 2.66, *p* < .01, as was the sex*gendercongruence interaction term β = − .454, *t*(479) = − 7.42, *p* < .001. The autism*gendercongruence interaction term was not a significant predictor β = .022, *t*(479) = .320, *p* = .75. The model at Step 2 explained a significantly higher proportion of the variance than the model at Step 1, *R*^2^ch. = .097, *F*ch. (3,479) = 26.27, *p* < .001. At Step 3, the three way interaction term was not a significant predictor of femininity, β = − .012, *t*(478) = − .173, *p* = .86, and the model did not account for a significantly higher proportion of the variance than at Step 2, *R*^2^ch. = .000, *F*ch. (1,478) = .03, *p* = .86.

Simple slopes analysis showed that there was a significant effect of having autism on femininity in females, with autistic females (*M* = 3.72, *SD* = .14) having significantly lower femininity than typically developing females (*M* = 5.14, *SD* = .11), β = − .413, *t*(482) = − 7.92, *p* < .001. There was no significant difference between autistic and TD males, with autistic males (*M* = 2.78, *SD* = .13) having equivalent femininity to typically developing males (*M* = 2.95, *SD* = .13), β = − .044, *t*(482) = − .820, *p* = .412 (see Fig. 2).

Simple slopes analysis revealed that there was a significant effect of sex on femininity in gender incongruent participants, β = − .415, *t*(482) = − 3.74, *p* < .001, with females (*M* = 3.07, *SD* = .35) reporting lower levels of femininity than males (*M* = 4.2, *SD* = .33). There was also a significant effect of sex on masculinity in gender congruent participants in the opposite direction, β = − .627, *t*(482) = 16.43, *p* < .001, with males (*M* = 2.75, *SD* = .09) reporting lower levels of femininity than females (*M* = 4.73, *SD* = .10).

## Discussion

The purpose of this study was to investigate sex differences in autistic and TD participants in social affiliation and attachment with a gender group (*gender identification*) and positive feelings derived from a gender group (*gender self-esteem*). There was support for the hypothesis that autistic people would identify less with and feel more negatively about a gender group as there was a significant effect of diagnostic group on both gender identification and gender self-esteem, with autistic participants scoring lower on both measures compared to TD controls. There was support for the hypothesis that gender incongruent participants would have lower gender identification and self-esteem than gender congruent participants. There was also support for the hypothesis that autistic females would have higher masculinity and lower femininity compared to TD females. Finally, there was support for the hypothesis that autistic natal females would have lower gender identification and gender self-esteem than controls, because while TD females had higher gender identification than TD males, the opposite pattern was found in the autism group, with autistic females having significantly lower gender identification than autistic males. There was no sex*autism interaction for gender self-esteem, but autistic participants had lower gender self-esteem than typically developing people, and females had lower gender self-esteem than males, and so autistic females had the lowest gender self-esteem. Also of relevance were the significantly lower rates of gender congruence, and higher rates of gender transition in all autistic participants, which were found to a greater extent in autistic females. The findings suggest that even gender congruent autistic people have lower gender identification and gender self-esteem than typically developing people.

We found that autistic natal females are particularly prone to lower social affiliation to a gender group, and greater variance in their gender expression, with these participants reporting lower femininity and higher masculinity than autistic males. There are two possible interpretations of this finding; the higher gender diversity in autistic natal females make it more difficult for this group to select a gender group to identify with, or they have selected a gender group and struggle to identify with other members of it. The finding that natal females are particularly affected fits with qualitative studies which found that autistic women often prefer to socialise with men (Bargiela et al. 2016). Research focusing on the lived experiences of autistic natal females regarding the development of their gender identity and feelings about gender groups would help to further explain these findings.

This study extends the previous findings regarding sex differences in gender identity in autism, demonstrating that the social component of gender identity is also affected in autistic people. This suggests that the higher variance in gender identities found in autistic people is associated with a lower sense of affiliation with and more negative feelings about gender groups as compared to controls.

There is increasing evidence that autism is a condition with high levels of gender variance (Bejerot and Eriksson 2014), and that gender identity is variant in autistic males as well as females (Bejerot et al. 2012; George and Stokes 2017). The current study replicated these findings, showing high levels of gender transition and incongruence in the autistic participants compared to TD participants. While not an objective of the study, we also found higher rates of diversity in sexual orientation in autistic compared to TD participants. Interestingly, autistic males considered themselves significantly less masculine than TD males, in line with previous findings (Stauder et al. 2011; Bejerot and Eriksson 2014). This would not be expected within the extreme male brain theory, however masculinity refers to the social norms associated with the gender expression of males, and it could be that autistic males rated themselves as lower in masculinity due to their awareness of being different to TD males. Masculine gender expressions may be seen as a marker of ‘typical’ social behaviour, and so autistic males may report lower levels of masculinity due to an awareness of their differences in social communication compared to other males. Therefore, according to these results both autistic females and significantly, males, appeared to display gender variance. The results of this study further suggest that autistic people are less likely to identify with a gender group, and see their gender groups more negatively than TD individuals. This is significant because gender identification and gender self-esteem are associated with psychological well-being in the TD population (Good and Sanchez 2010; Sanchez and Vilain 2009). Furthermore, there is evidence that gender dysphoria is associated with poor mental health in autistic people (George and Stokes 2018), and social identity processes are relevant to psychological well-being in autistic people (Cooper et al. 2017), and so the low gender identification and self-esteem found in autistic participants could impact on quality of life in this group. However, we did not measure psychological well-being in this study and cannot draw conclusions regarding the link between gender identification and well-being. An alternative hypothesis is that autistic people are less constrained by the gender norms of the typically developing population, and the high levels of gender diversity in autistic people are in fact associated with improved mental health. Further research is needed to unpack the relationship between gender identification, gender self-esteem and psychological well-being in the autism community.

There are features of autism which may contribute to this greater rate of gender diversity and lower gender identification across the sexes, however there is limited research available about how autistic individuals think about gender as a concept as compared to typically developing individuals, and so the following theories may or may not relate to gender identity in this group. Deficits in self-categorisation may pose challenges to autistic individuals in identifying with a gender label (Skorich et al. 2016), as individuals who struggle to place themselves in social categories may develop more idiosyncratic gender identities compared to typically developing people who are more readily able to categorise themselves within a gender group. Deficits in social communication may result in less knowledge and understanding of gender norms, or a freedom from gender norms allowing for more diverse gender expression, and a lower level of social reciprocity and affiliation with members of a gender group. Biological factors may also play a part, with factors such as prenatal testosterone linked to the development of autism and gender diversity (Baron-Cohen et al. 2005). Furthermore, for autistic people who have non-traditional gender identities, accessing and socialising with minority gender groups may be a particular challenge, as these are smaller and therefore less accessible groups. Indeed, George and Stokes (2017) found significant associations between all subscales of the Autism Quotient (AQ) and a measure of gender dysphoria, the strongest associations being with the social and communication subscales of the AQ. This suggests that the social communication deficits present in autism may contribute to differences in gender identity, or vice versa, the experience of gender dysphoria could contribute to social difficulties. This fits with the current findings that social identification with a gender group was lower in autistic participants compared to controls. Furthermore, gender self-esteem is likely to be lower if one belongs to a group which is stigmatised by society, and unfortunately gender variant individuals are more likely to be bullied (Russell et al. 2011) and ostracised (Carter and McCloskey 1984). Autistic people are also at higher risk of being bullied than typically developing people (Schroeder et al. 2014), and so an autistic person with gender dysphoria must contend with two stigmatised identities. Further research is needed into the experiences of autistic people with a range of gender identities, to investigate how their gender identity developed in relation to others of the same or different genders, and how this has impacted on quality of life.

Our findings also show that autistic natal females had significantly more diverse gender identities compared to autistic males. Furthermore, they tended to identify less strongly with a gender group than autistic males. It was noteworthy that the higher gender identification for TD females as compared to TD males found in this study was inverted for the autistic females, who had lower gender identification than autistic males. The extreme male brain theory has highlighted biological factors related to the development of autism and the male sex, for example the high levels of prenatal testosterone in autism (Baron-Cohen et al. 2005). It is possible that these biological factors impact on the gender identity of females. There were high levels of gender incongruence in autistic natal females compared to males. However, autistic females who were not gender congruent were much more likely to identify outside of a gender binary, with just 7% identifying as male and 26.5% identifying with a non-binary ‘other’ identity. This suggests that while it is possible that biological factors associated with autism may impact gender identity in females more than in males, this does not lead to a higher masculine gender identity, but to individuals identifying outside of the gender binary. This is despite autistic females rating themselves as significantly more masculine than TD females.

One limitation of this study was the possible bias in the sample. Participants self-identified as having a formal diagnosis of autism, and so it is possible that some participants did not have a diagnosis from a medical professional. Furthermore, the average age of ASD diagnosis was in early adulthood, and there was an unusually high rate of gender and sexual orientation diversity and mental health problems in both the autism and TD sample, suggestive of selection bias. There was a discrepancy in mental health diagnoses between autistic and TD participants; this may in part be related to differences in rates of gender dysphoria between these groups (George and Stokes 2018). Some clinicians have posited that there may be some false diagnoses of autism in those with gender dysphoria due to high levels of social anxiety in this group; it was beyond the scope of this research to investigate this. Another limitation relates to the gender identification and self-esteem measures. We asked participants to respond to items referring to their relationship with and thoughts about their “gender group”, and this was left open to their interpretation. Therefore for those participants who were in the process of transitioning between gender groups, it is not clear which gender group they responded for. However, gender congruence was included in the analysis as an independent variable, and we still found that the sex*autism interaction term was a significant predictor in three of four analyses. Furthermore, social affiliation to any gender group is of interest, given the findings that belonging to a wide range of social groups has positive effects on physical and psychological well-being in the typically developing population (Jetten et al. 2012), and that affiliation with other autistic people is associated with higher psychological well-being in autistic people (Cooper et al. 2017). However, the relationship between affiliation to gender groups and well-being in the autism community is unknown and would benefit from further investigation.

A strength of this study is its focus on the social processes related to, but distinct from gender identity, specifically gender identification and gender self-esteem, which to the authors’ knowledge have not yet been quantitatively investigated in autism. Furthermore, many autistic participants opted to describe their gender using the ‘other’ option, revealing that autistic people frequently identified outside of a gender binary, an option which previous surveys have not reported.

Given evidence of differences in gender identity in this group, it is important to consider the way that autistic people are supported with gender issues. Health services are likely to come into contact with autistic people who are gender diverse for numerous reasons, for instance for physical health care, mental health care, or for support around gender identity and transition. It is important that healthcare professionals working with this group are able to make reasonable adjustments to the care they give this group to ensure that treatment is accessible and effective. Furthermore, psychosocial interventions are likely to be useful for a group who have a lower sense of social affiliation to gender groups. One such intervention would be peer support groups of gender diverse autistic people where group members can share their stories and experiences of being autistic and gender diverse and support other group members. Future research should prioritise the voices of individuals who are members of both the autism and gender diverse communities through qualitative research exploring their experiences of belonging to these groups, and outcome research investigating the efficacy of psychosocial support groups and gender specific interventions for this group.

In sum, this study explored the social aspects of gender identity in autistic people, focusing on gender identification and gender self-esteem. The results corroborate those of previous studies which found high rates of gender variance in autism, with females particularly varied in their gender identities. It further extends these findings, suggesting that there are sex differences within the autism population, different to those found in the typically developing population, and that autistic individuals have weaker identification with gender groups, and feel less positively their gender groups than TD controls.

## Footnotes

1 There is evidence that the sex differences found in empathising and systemising skills in typically developing people may be modulated by cultural norms and motivation, rather than being hard-wired sex differences (e.g., Nosek et al. 2009; Klein and Hodges 2001). However the fact remains that in most Western cultures these traits are perceived to be related to sex. Therefore, in Western cultures, both autistic males and females may be perceived by others as having more ‘male’ characteristics.
